# Recent Advances in the Development of Hybrid Drugs

**DOI:** 10.3390/pharmaceutics16070889

**Published:** 2024-07-01

**Authors:** Tânia S. Morais

**Affiliations:** 1 Centro de Química Estrutural, Institute of Molecular Sciences, Faculdade de Ciências, Universidade de Lisboa, Campo Grande, 1749-016 Lisboa, Portugal; tsmorais@ciencias.ulisboa.pt; 2 Departamento de Química e Bioquímica, Faculdade de Ciências, Universidade de Lisboa, Campo Grande, 1749-016 Lisboa, Portugal

In the search for innovative, selective, effective, and safer treatment strategies, hybrid drugs have gained worldwide momentum. Nevertheless, the concept of hybrid drugs is not entirely new. Early examples include combination therapies where two or more drugs were co-administered. The hybrid drug approach is a sophisticated form of combination therapy that can be particularly valuable in treating complex and multifactorial conditions such as cancer [1,2,3,4,5], infectious diseases [6,7,8,9,10], and neurological disorders [11,12,13,14] where traditional single-target therapies often fall short.

Molecular hybridization is a rational drug design strategy that combines two or more covalently binding pharmacological agents into a single multi-functional molecule or formulation [15,16,17]. Such hybrids must maintain the structural features, activity and affinity to the specific targets of the original drugs [2,16]. The presence of two or more components may act synergistically or complementarily to target multiple pathways or mechanisms within the body associated with a particular disease or condition. As hybridized fragments share the same pharmacokinetic profiles, this molecular design approach ensures that the required concentrations of each pharmacophore are available near the target(s) at the exact same time, lowering the required doses—something that is often crucial for optimal results [1,2]. Thus, these drugs are designed to enhance therapeutic efficacy, minimize side effects, and overcome limitations associated with conventional classical therapies, such as drug resistance by amplification or exerting multifactorial biological activities [18,19,20].

The development of hybrid drugs presents several significant challenges, and these include the complexity of their design and synthesis, their pharmacokinetic and pharmacodynamic profiles, and the costs associated with the development of more complex drugs. However, their undeniable advantages over conventional therapies has led several researchers to integrate molecular hybridization into strategic drug development approaches for advanced drug delivery systems and targeted/precision therapy. This has been reflected by an increase in the number of relevant publications in the field and the increasing number of hybrid drugs that have successfully reached the market or are in advanced clinical trial stages [2,21,22,23,24,25].

This Special Issue highlights several recent developments and emerging trends in the field of hybrid drugs. This issue comprises four original research articles and three reviews that explore their design and synthesis and structure–activity relationships, as well as mechanistic studies of biological targets and pathways. It also addresses significant applications, challenges, and opportunities in the field.

Based on the promising individual properties of resveratrol and hydrazones as potential anticancer agents, Castrillón-López et al. (contribution 1) designed a family of resveratrol–hydrazone hybrids, aiming to create improved dual-effect compounds against colorectal cancer. In this publication, the authors present a synthesis, in vitro evaluation of cytotoxicity against human colorectal cancer cells (SW480 and Sw620) and normal cell lines (HaCaT and CHO-K1), as well as a preliminary assessment of the cell death mechanism of these new entities. The authors were able to find two lead hybrids that showed improved activity and selectivity for colorectal cancer compared with the resveratrol and hydrazone counterparts.

In their publication, Pele et al. (contribution 2) present a synthesis, in vitro evaluation of the antioxidant and cytotoxic activity of polyphenolic-quinazolin-4(3H)-one hybrid drugs. Quinazoline/quinazolinone moieties are among the most important wide-range pharmacophores with anti-HIV, anticancer, antifungal, antibacterial, anti-inflammatory, antimalarial, antioxidant, and antileishmanial properties [26,27,28,29]. In a previous work, the authors enhanced the antiradical activity of quinazolin-4(3H)-one when linked to antioxidant phenolic derivatives through a thioacetohydrazone linker. In this publication, the authors explored the influence of two structural derivatizations in the antioxidant activity of these hybrid drugs, namely the introduction of an additional phenolic group and the insertion of an electron-donating group on the arylidene carbon linked to the polyphenolic moiety.

The ineffectiveness and failure of chemotherapeutic treatments are often linked to multidrug resistance (MDR), which is primarily associated with the overexpression of ATP-binding cassette (ABC) transporter proteins in cancer cells [30,31,32]. The quinazoline scaffold has been reported as the core structure of several ABC inhibitors; however, their negative effects include their intrinsic toxicity and metabolic instability. Driven to surpass these limitations, Stockmann et al. (contribution 3) developed a series of carbonyl 2-phenylquinazoline hybrid drugs combining the ABC inhibitor properties of 2-phenylquinazoline with the non-toxic and metabolic, stable, three-dimensional carborane pharmacophore to inhibit ABC transporters and reverse MDR. In this publication, the authors explored several chemical, computational, and biological methods to determine the potential of these hybrid drugs in the targeted inhibition and reversion of ABC-mediated MDR.

The prodrug tamoxifen is widely used in clinical practice as a selective estrogen receptor modulator for the treatment of estrogen-receptor-positive breast cancer (ERα), but the development of resistance limits its effectiveness [33,34]. Over the years, tamoxifen’s structure has been successfully modified to be combined with several transition metals, such as iron, ruthenium, osmium, copper, and platinum, among many others [35,36,37,38,39]. The most renowned/successful was the Ferrocifen hybrid molecule—a tamoxifen combination with ferrocene [40]. Kazimir et al. (contribution 4) reported an innovative approach based on combining a tamoxifen derivative with the 2,2′-bipydine ligand—a ligand widely used with metal complexes for medicinal applications—and its complexation to Pd and Pt complexes. In this publication, the authors reported the synthesis and biological evaluation of these new promising hybrid drugs.

Tiz et al. (contribution 5) provide an overview of hybrid drugs recently approved by the FDA in 2022. Many of these drugs that feature active moieties that the FDA had not previously approved, whether as single ingredients or as components of combinations, offer important new therapies for patients with unmet medical needs. In this review article, the authors discuss the synthesis and therapeutic effects of diverse small molecules ranging from classical chemical scaffolds to more innovative drugs.

Zlotos et al. (contribution 6) review the advancements made from 2010 to 2022 in incorporating estrogen receptor (ER) ligands into anticancer hybrid drugs. In particular, the authors discuss the design, structures, and therapeutical effects of a series of hybrid conjugates that include ER ligands such as selective ER modulators (tamoxifen, 4-hydroxytamoxifen, endoxifen), selective ER degraders (ICI-164384), and ER agonists (estradiol). These ligands are linked to various anticancer agents, including histone deacetylase inhibitors, DNA-alkylating agents, antimitotic agents, and epidermal growth factor receptor inhibitors. In this review article, the authors also demonstrate the great potential of ER ligands as carriers for drug delivery.

Hurwitz et al. (contribution 7) summarize the recent advances of the antibody–drug conjugates (ADCs) designed for targeted cancer therapy and discuss their mechanisms of action, therapeutic potential, key trials, approved indications, and common themes. In this review article, the authors also highlight the current challenges and opportunities faced by this drug class amidst the rapid advancements in antibody therapies, immunotherapy, and targeted cancer agents.
